# Understanding transitional care programs for older adults who experience delayed discharge: a scoping review

**DOI:** 10.1186/s12877-021-02099-9

**Published:** 2021-03-29

**Authors:** Katherine S. McGilton, Shirin Vellani, Alexandra Krassikova, Sheryl Robertson, Constance Irwin, Alexia Cumal, Jennifer Bethell, Elaine Burr, Margaret Keatings, Sandra McKay, Kathryn Nichol, Martine Puts, Anita Singh, Souraya Sidani

**Affiliations:** 1 KITE-Toronto Rehabilitation Institute, University Health Network, 550 University Avenue, Toronto, Ontario Canada; 2 Lawrence S. Bloomberg, Faculty of Nursing, University of Toronto, Toronto, Ontario Canada; 3 Rehabilitation Sciences Institute, Faculty of Medicine, University of Toronto, Toronto, Ontario Canada; 4 Care Transitions, Health Sciences North, Sudbury, Ontario Canada; 5 Visiting Homemakers Association Home Healthcare, Toronto, Ontario Canada; 6 Dalla Lana School of Public Health, University of Toronto, Toronto, Ontario Canada; 7 Ontario Ministry of Health and Long-Term Care, Toronto, Ontario Canada; 8 Daphne Cockwell School of Nursing, Ryerson University, Toronto, Ontario Canada

**Keywords:** Transitional care programs, Delayed discharge, Aged, Scoping review, Structure, Components, Outcomes

## Abstract

**Background:**

Many hospitalized older adults cannot be discharged because they lack the health and social support to meet their post-acute care needs. Transitional care programs (TCPs) are designed to provide short-term and low-intensity restorative care to these older adults experiencing or at risk for delayed discharge. However, little is known about the contextual factors (i.e., patient, staff and environmental characteristics) that may influence the implementation and outcomes of TCPs. This scoping review aims to answer: 1) What are socio-demographic and/or clinical characteristics of older patients served by TCPs?; 2) What are the core components provided by TCPs?; and 3) What patient, caregiver, and health system outcomes have been investigated and what changes in these outcomes have been reported for TCPs?

**Methods:**

The six-step scoping review framework and PRISMA-ScR checklist were followed. Studies were included if they presented models of TCPs and evaluated them in community-dwelling older adults (65+) experiencing or at-risk for delayed discharge. The data synthesis was informed by a framework, consistent with Donabedian’s structure-process-outcome model.

**Results:**

TCP patients were typically older women with multiple chronic conditions and some cognitive impairment, functionally dependent and living alone. The review identified five core components of TCPs: assessment; care planning and monitoring; treatment; discharge planning; and patient, family and staff education. The main outcomes examined were functional status and discharge destination. The results were discussed with a view to inform policy makers, clinicians and administrators designing and evaluating TCPs as a strategy for addressing delayed hospital discharges.

**Conclusion:**

TCPs can influence outcomes for older adults, including returning home. TCPs should be designed to incorporate interdisciplinary care teams, proactively admit those at risk of delayed discharge, accommodate persons with cognitive impairment and involve care partners. Additional studies are required to investigate the contributions of TCPs within integrated health care systems.

**Supplementary Information:**

The online version contains supplementary material available at 10.1186/s12877-021-02099-9.

## Background

Many older adults live with multiple complex health problems or experience unforeseen circumstances (e.g., falls) that result in acute care hospital admissions. Among hospitalized older adults, some cannot be discharged once intensive medical treatments are completed because they lack sufficient health and social supports (e.g., community services, nursing home beds) to meet their post-acute care needs [1]. Delayed discharges—episodes where patients have to stay in an acute care bed for longer than medically necessary—present critical challenges for many health care systems [2]. In Canada, patients who experience delayed discharge are referred to as Alternate Level of Care (ALC), and they occupy 8 to 10% of acute care hospital beds [3].

Delayed discharges are an important health issue on both an individual and health system levels. McCloskey and colleagues [4] found that mean age of this group was 79 years; 65% were women; had a mean of 4.6 chronic conditions (mainly hypertension, heart disease and diabetes); and were on multiple medications. During their hospital stay, most showed functional decline (reduced mobility and ability to perform basic activities of daily living); had inadequate home support both prior to admission and following discharge from hospital; and were waiting for transfer to a long-term care facility [5]. These older patients often developed complications (e.g., poor oral intake, confusion, infections), felt socially isolated [1] and, alongside their care partners, reported increased depressive [3], anxiety, and stress symptoms [5, 6]. Also, some hospitals charge patients with a daily co-payment for prolonged delayed discharge, which may contribute to a substantial financial burden on individuals and families [6]. At the system level, delayed discharges reduce patient flow, leading to emergency department overcrowding, cancelled elective outpatient surgeries, and poor coordination of care resources across healthcare settings [1, 3].

To address these issues, healthcare systems globally have introduced programs to provide primarily restorative care, to older patients either experiencing, or at risk for, delayed discharge. These programs serve as a means to maintain continuity of care in the interface between acute care and other settings such as residential care or an individual’s home. These programs include Intermediate Care Models (ICMs) in the UK and Europe; sub-acute care, post-acute care or skilled nursing facilities (SNFs) in the US; and Transitional Care Programs (TCPs) in Australia and Canada, which will all hereafter collectively be referred to as TCPs. TCPs are designed to provide short term, low intensity, restorative care to older adults (aged 65+) who are medically fit to leave the hospital but are unable to do so due to multiple issues including hospital acquired deconditioning and lack of social supports in the community [3]. Differences in TCPs across countries makes it difficult to describe and compare the patient populations they serve, the services they offer, the resources required to deliver them, and their expected outcomes [7]. To address this concern, Sezgin et al. [8] identified and defined the characteristics of ICMs based on the results of a scoping review and an international expert consultation (Delphi study and consensus meeting); the study identified key defining features of ICMs, including time-limited care delivered by interdisciplinary teams that promote recovery, restore independence and act as the interface between acute care services and home. In a subsequent review focused on the effectiveness of ICMs, Sezgin et al. [9] found that while some researchers reported reduced hospital utilisation, evidence was limited on the ICMs’ effectiveness in reducing emergency readmissions, institutionalization and cost, or improving older adults’ function. The inconsistent findings may be attributable to contextual factors (i.e., characteristics of patients, staff and environment) that may influence the implementation and outcomes of TCPs [10]. Contextual factors may impact the actual delivery of the TCPs’ core components which are aimed at reducing and preventing delayed hospital discharges in older adults, and with achieving best outcomes for this population.

This scoping review was conducted to describe contextual factors and core components of TCPs, and outcomes used to evaluate TCPs. The review was informed by Donabedian’s structure-process-outcome framework [11]. Structures are defined as attributes or characteristics that are within the health care team, organization, or patient that influence the implementation of the core components or services and the outcomes of TCPs. For example, in the current context, the patient structure covers the characteristics of older patients admitted to TCPs, and the resources (i.e., healthcare team) needed for providing high-quality services. Processes represent the core components and specific services offered, as well as the behaviours or actions reflecting collaboration between the individuals and their surroundings. For TCPs, examples of processes include the range of physical and psycho-social services provided by the TCP staff. Potential outcomes targeted at patients include functional status for patients, and the healthcare system outcomes such as discharge destinations, as healthcare system outcomes. Specifically, the purpose of this review was to identify research evidence and knowledge gaps related to the structures, processes and outcomes of TCPs. The specific review questions were:

1. What are the socio-demographic and clinical characteristics of older patients served by TCPs?
2. What are the core components provided by TCPs?
3. What patient, caregiver, and health system outcomes have been investigated and what changes in these outcomes have been reported for TCPs?

## Method

A scoping review (ScR) is the most suitable method for knowledge synthesis to address our exploratory research questions [12]. We followed the ScR methodology framework outlined by Arksey and O’Malley [13] and refined by Levac et al. [14], Colquhoun et al. [12], and Daudt et al. [15] as outlined in the published study protocol [7]. We followed the Preferred Reporting Items for Systematic Review and Meta-Analysis Protocols extension for ScRs statement (see Additional file 1). The ScR framework includes six steps: 1) identifying the research questions (listed above); 2) identifying relevant literature; 3) study selection; 4) charting the data; 5) collating, summarizing and reporting the results; 6) consulting with key stakeholders and translating knowledge. Below we briefly summarize each step.

### Search strategy

Based on the descriptive aims of the review, the following key words and Medical Subject Headings (MeSH) terms were included in the search to identify relevant literature: aged or Indigenous persons and transitional care programs or intermediate care facilities or post or sub-acute facilities. We included Indigenous persons in our search to capture programs that might serve their unique needs and to help guide effective planning in this population [16]. A comprehensive search strategy was developed in conjunction with a health sciences librarian and subsequently refined in consultation with stakeholders and the research team. The strategy was validated by ensuring the retrieval of a key set of relevant studies. Searches were conducted in MEDLINE(R) (in Ovid, including Epub Ahead of Print, In-Process & Other Non-Indexed Citations, Ovid MEDLINE(R) Daily), Embase (in Ovid, including Embase Classic), Emcare (Ovid), PsycINFO (Ovid) and CINAHL (in EBSCO). All searches were conducted on May 28th, 2019 and updated on January 3rd, 2020. Search strategies included the use of text words and subject headings (e.g., MeSH, Emtree) related to (1) alternate levels of care, sub-acute, SNF, (2) aged populations and (3) Indigenous populations. Searches were limited to descriptive and evaluative studies of TCPs targeting older adults (55+), and reported in English, French, Dutch and German languages, when possible. For a more comprehensive search narrative and Medline search strategy, see Additional file 1 for the full Medline search strategy.

### Study selection

**Fig. 1:**
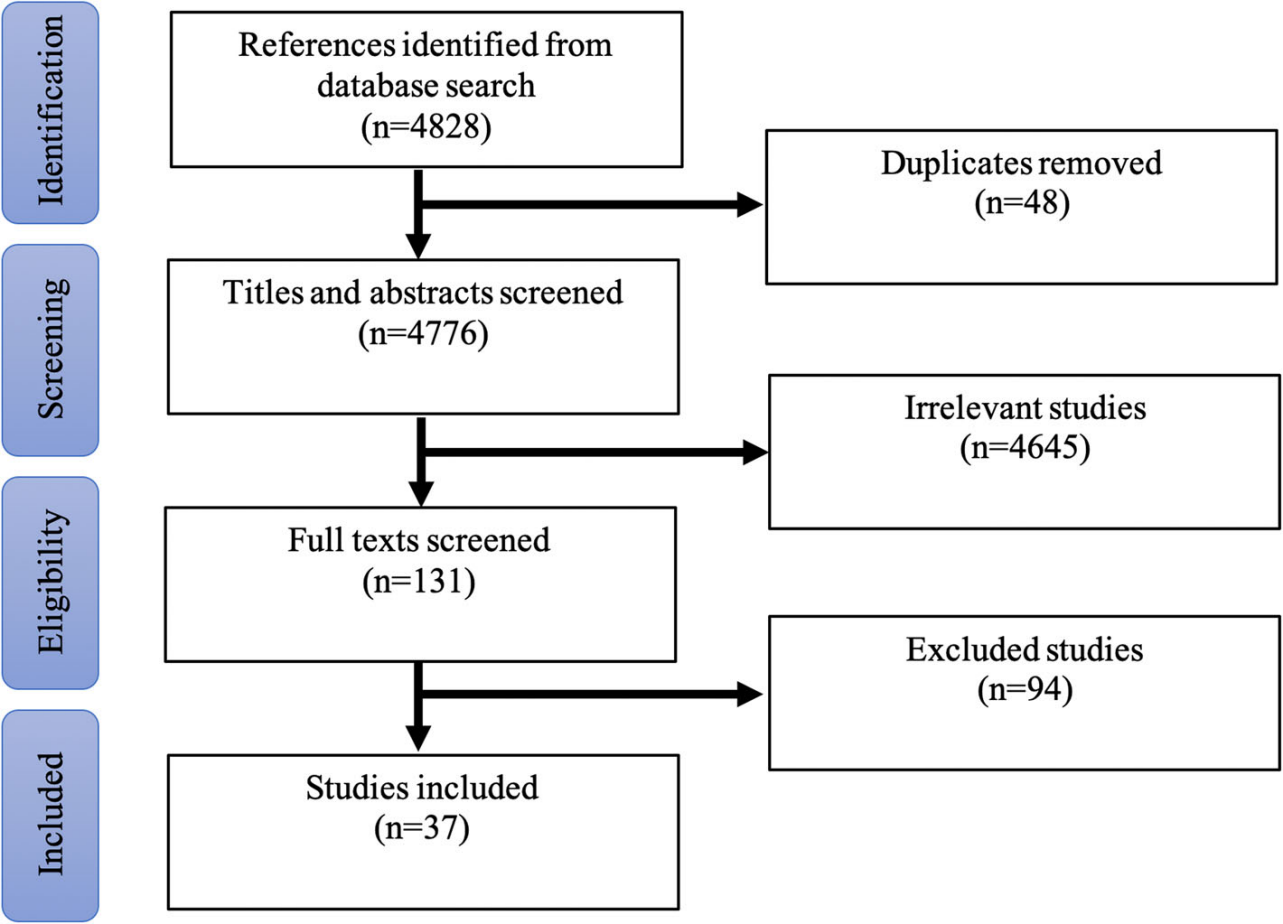
PRISMA Flow Diagram

Titles and abstracts were independently reviewed by two researchers against the selection criteria below. Then, two reviewers independently assessed all potentially relevant full text articles against inclusion and exclusion criteria (see Fig. 1 PRISMA flow diagram). In cases of disagreement between reviewers, the principal investigator (KM) resolved the conflict.

Studies were eligible for inclusion if they reported models of TCPs that served community-dwelling older adults (aged 65 years or older) experiencing or at risk for delayed discharges and if they reported examining processes and/or outcomes used to evaluate the programs. Studies that focused on standards of care in transitioning from acute care to home (e.g., rehabilitation post fracture or stroke) or involved home-based TCPs were excluded.

### Data abstraction

A data abstraction form was created with specific codes based on Donabedian categorizations. The form was pilot tested by two members of the study team (KM, SV) to ensure clarity of content and consistency in documentation. Data extracted included study characteristics (i.e., type of study or design, aim, country, target population, sample size, setting, response rate); patient characteristics (age, sex, ethnicity, location, number and types chronic conditions); TCP features (location, staff mix, goals, patient admission criteria, and core components or services provided); and outcomes investigated. We used TIDier checklist to abstract data on elements of the TCPs reported in the selected articles [17].

Finally, we organized a stakeholder consultation meeting on 24 February 2020. Participants included representatives of: family caregivers (*n* = 1), health care practitioners (*n* = 3), hospitals (*n* = 6), community support service agencies (*n* = 5), provincial health care policy organisations (*n* = 4), local health regions (*n* = 2), and private service providers (*n* = 2). They provided feedback on the results, which are summarized below.

## Results

### Characteristics of included studies

Table 1 provides a summary of the studies selected for review. Although we aimed to include evidence involving Indigenous persons, we did not find any applicable research. Forty-nine percent of the studies were conducted in Europe [18, 19, 29–33, 35–39, 42, 43, 45, 51, 53, 55, 57], 27% in the U.S.A. [20–23, 25, 26, 44, 52, 54, 56], 11% in Australia [24, 27, 28, 48], 8% in Asia [40, 41, 49], and 5% in Canada [46, 50]. Different study designs were used, with the most common being retrospective cohort or chart audit designs (*n* = 9) [20, 21, 25, 27, 30, 39, 46, 49, 52]; followed by prospective cohort design (*n* = 7) [18, 19, 24, 26, 41, 44, 45]; RCTs (*n* = 7) [23, 28, 33, 35, 38, 53, 55]; non-randomized design (*n* = 4) [22, 29, 40, 57]; case study (*n* = 2) [31, 32]; pre-test post-test (*n* = 2) [48, 50]; and other designs (*n* = 6) [37, 42, 43, 51, 54, 56].

**Table 1:** Summary of Included Studies - Patient, Staff and Setting Characteristics

First Author, Publication Year Region	Design	Sample Size	Setting	Staff Mix	Sample Characteristics (At the time of admission to TCP)					
					Age [Mean (Range or SD)]	Female (%)	Multimorbidity [%, Mean (Range or SD)]	Cognitive Status [Mean (Range or SD)]	Function [Mean (Range or SD)]	Living Arrangement
Abrahamsen, 2014 [18] Europe	Prospective cohort study	557	LTCH/NH	Geriatrician, physician, nurses, PT & OT	Median: 86	70	61%	MMSE: mean: 26 (8–30)	BI: 15 (4–20)	74% alone
Abrahamsen, 2016 [19] Europe	Prospective, cohort study	961	LTCH/NH	Nursing, OT, PT, SW & dietitian	85	68.4	59%	MMSE: 26 (8–30)	BI: 75 (10–100)	67% alone
Anderson, 2005 [20] US	Retrospective, medical record audit	68	Community hospital	Nursing, PT, OT, SLP, dietitian. If needed, respiratory therapy	79.7	64.7	Majority had 8 conditions	Most patients oriented to the environment; 50% had intact STM	NR	26% alone 57.3% with someone 7.4% seniors housing
Bergman-Evans, 2010 [21] US	Retrospective cohort study	1430	SNF	NPs, physicians, SW,	78	64	Average # of medications: 10.1	NR	NR	NR
Blewett, 2010 [22] US	Multi-method non-randomized experimental study	339	SNF	Geriatrician, geriatric NP, geriatric pharmacist, nursing, dietitian, SW, therapeutic recreation & spiritual care	76.8	66	NR	NR	NR	NR
Bowcutt, 2000 [23] US	Randomized controlled trial	191	Subacute unit inside University hospital	Geriatric CNS, nurses, dietitian, PT, OT, SLP, hearing therapist, SW, activity specialist, chaplain, physicians.	72.9	61.8	NR	Had to be mentally alert	NR	NR
Brusco, 2012 [24] Australia	Prospective cohort study	696	Hospital ward, residential care facility, Community i.e., patients’ homes	PT, physicians, nursing & other allied health and support services staff	81.9 (8.7)	59.5	CCI: 1.7 (1.8)	NR	BI: 60.4 (28.9)	NR
Burke, 2015 [25] US	Retrospective survey analysis	2.99 million	SNF	NA	76.7	63	NR	NR	NR	NR
Buurman, 2016 [26] US	Prospective cohort study	394	SNF	Rehabilitation staff-not clearly reported	84.9	67.8	Had 2 or more medical conditions.	MMSE < 24: 25.6%	No. of disabilities: 6 (2–9)	48.2% alone
Chan, 2019 [27] Australia	Retrospective cohort study	369	LTCH/NH	Geriatrician, RN, PT, OT & SW	82.9 (SD 7.84)	64.80	CCI: 2.5 (2.1)	23.6% had Cognitive impairment; 10% had dementia diagnosis	Baseline BI Mean (SD): 64.0 (58.7)	58.3% alone
Crotty, 2005 [28] Australia	Randomized controlled trial	317	Transitional care facility	Pharmacist, geriatrician, rehabilitation medicine physician, PT, SW, GP, nursing, other allied health staff & a transitional care nurse coordinator	83.0 (7.2)	49	NR	NR	BI: CG - 50.5 (29.7); IG - 45.8 (30.7)	NR
Dahl, 2015 [29] Europe	Nonrandomized observational study	328	Intermediate care hospital	Nurses, OT, PT & physician	75.5	56.5	4.2 (2.0)	NR	17 ADL TCP: 1.95 (0.74) CG: 1.95 (0.82)	NR
DelGiudice, 2009 [30] Europe	Retrospective cohort study	375	Acute care ward	Physicians (geri, neuro, rehab, surgery and other specialists); nursing, PT, SLP, dietitian, SW & nurses	80.5	51.7	22.5%: 0–2 conditions; 73.5%: 3–5; 4.1%: 6–8 conditions	Moderate/severe: 16.3% Low/none: 83.7%	Katz (ADL): 63.3% dependent; 36.7% self-sufficient Lawton (IADL): Dependent: 65.1 Self-sufficient: 31 No data: 4	NR
Dixon, 2010 [31] Europe	Case study	403	Intermediate care	PT, OT, and therapy assistants, with a large range of other staff that varies greatly.	Median: 82.14	74.2	NR	NR	BI: 15.00 (3–20)	NR
Elbourne, 2012 [32] Europe	Case study	*n* = 94 service users *n* = 12 staff	20 bed facility within a total care living complex	Nurses, care workers, PT, OT, SW & doctors	NR	NR	NR	NR	NR	NR
*Garåsen, 2007 [33]; Garåsen, 2008 [34] Europe	Randomized controlled trial	142	LTCH/NH	trained nurses, general practitioners	80.6	72.2	NR	NR	ADL Score: TCP: 2.24 (0.9); CG: 2.05 (0.7)	22% lived with spouse
*Griffiths, 2001 [35]; Harris, 2005 [36] Europe	Randomized controlled trial	176	General hospital	PT, OT, NPs, nurses, clinical nurse specialists, chiropodist, doctors, dietician, SLP, SW.	78.3	67	NR	NR	Barthel index BI = 12.3	NR
Heim, 2016 [37] Europe	Action research project	1933	Community hospital and nursing home	Multidisciplinary team including geriatrician, geriatric trained nurses and physiotherapists	78.5	52.95	NR	NR	ADL limitations prior to admission frail: 36.9; 29.5 non-frail: 3.3; 3.9	NR
Herfjord, 2014 [38] Europe	parallel group Randomized controlled trial	376	LTCH/NH	physician (specialist in geriatric medicine and internal medicine/junior doctor supervised by a geriatrician), skilled nurse, physiotherapist, health care worker	83.6 (70–96)	73.2	NR	no severe dementia, delirium (but staff not specified how to assess delirium or dementia)	BI = 70.0 medical: 80.0 ortho: 56.7	NR
Hilton, 2013 [39] Europe	Retrospective cohort study	100	LTCH/NH	Nursing, staff psychiatrist, PT, OT, psychologist. Visiting senior medical support included a GP, a psychiatrist, and a geriatrician	82.6	65	NR	NR	NR	NR
Lee, 2011 [40] Asia	Non-randomized intervention cohort study	245	Community hospital	“Interdisciplinary geriatric team”	82.7 (5.5)	NR	NR	MMSE: 13.4 +/− 8.1	BI: 47.1 +/− 33.6 Lawton-Brody IADL: 2.1 +/− 2.4	NR
Lee, 2012 [41] Asia	Prospective cohort study	83	Community hospital	Interdisciplinary team	80.3	3.60%	NR	Persons with severe dementia excluded	BI 35.0 +/− 21.3	59.3% alone
Lees, 2013 [42] Europe	Mixed methods triangulation approach	9	Purpose built interim (transitional) care unit	Nurses, geriatrician, health care assistants	75.1	40%	3–8	NR	NR	NR
Levin, 2019 [43] Europe	Quasi experimental, interrupted time series	107,022	LTCH	Care home providers, SW, hospital ward staff, GP, community teams of allied health professionals, social housing associations	81.7 (5.7)	64.5	NR	NR	NR	NR
Likourezos, 2002 [44] US	Prospective cohort study	164	LTCH/NH	Physicians, ethics consultation team, radiology, orthopedic, dental, eye, and other specialty clinics, PT, OT, SLP	81 (61–103)	76.8	CCI: 1.0 (0–4)	FIM Cognitive score 30 (range: 9–35)	FIM Motor score 46.5 (13–78)	NR
Luthy, 2007 [45] Europe	Prospective cohort study	166	General medicine ward	Physicians, nurses, psychiatrists, psychologist, PT, OT & SW	74.6 (15.6)	52	CCI: 3.6 (1.9)	NR	Number of ADL impairment: 2.0 (1.9) Number of IADL impairments: 2.8 (1.5)	55% alone
Manville, 2014 [46] Canada	Retrospective chart review	135	Acute care hospital	Nursing, PT/OT, rehabilitation aides, nutritionist, pharmacist. FPs (with care of the elderly training),	86.4	63	CCI: 3.16	51% had dementia diagnosis	NR	55% alone
*Michael, 2005 [47]; Michael, 2004 [48] Australia	Pre-test post-test	168	Acute care hospital	Nurses, therapy assistants, geriatrician, allied health services: pharmacy, PT, OT, SW, SLP & clinical psychology	81.38	84	NR	Persons with dementia and severe CI were excluded	BI: 87.3 (12.7)	51.8% alone 45.8% with others
Nakanishi, 2016 [49] Asia	Retrospective cohort study	9992	Acute care hospitals, community hospitals, LTCH/NH & patients’ own homes	NR	85.4	70.5	NR	Dementia: 24.8%		NR
O’Brien, 2017 [50] Canada	Pre-test post-test	49	LTCH/NH	physician, OT, PT, SW, dietician, nurse, & recreation specialist	83 (66–96)	71.4	Moderate to severe cognitive impairment Frequent, uncontrolled inappropriate behaviors were excluded	NR	BI: 54.20 (SD = 19.26)	NR
Orvik, 2016 [51] Europe	Retrospective and prospective multimethod observational study	88	LTCH/NH	nurses, physician & PT	NR	NR	NR	Persons with dementia excluded	NR	NR
Parsons, 2002 [52] US	Retrospective chart review	154	Acute care hospital	Physicians, nurses, pharmacists, SW, PT, OT & NPs	76	3	Average 4 comorbidities	NR	40% dependent in all ADLs; whereas 26% independent in all ADLs.	62% lived with caregivers
Richardson, 2001 [53] Europe	Randomized controlled trial	276	Acute care hospital	Nurses, GP, OT/PT, SLP, dietician, SW, pain team, CNS	76	61	NR	NR	NR	NR
Richardson, 1986 [54] US	Mixed/multiple methods	NR	Rural hospital	OT, PT, SLP, Hearing services, dental, SW, discharge planners	75+	NR: mostly women	NR	NR	NR	~ 50% lived alone
Steiner, 2001 [55] Europe	Randomized controlled trial	240	Acute care hospital	Nurses, PT, doctors can be called in an emergency	72.2	49	NR	Cognitively impaired at randomisation intervention: 39/114; control 32/119	BI: IG 63.5 (23.5); CG 60.3 (22.8)	62.4% alone
Tappen, 2001 [56] US	Quasi-experimental repeated measures	242	SNF	Nursing, other professions NR	76.8 (SD 11.18)	74	1.92	Excluded if MMSE < 21	FIM: 84.34 (21.76)% Katz index: 6.32 (2.89) Lawton & Brody scale: 10.73 (4.80)	NR, but eligible if adequate cognition
Trappes-Lomax, 2006 [57] Europe	Non-randomized controlled trial	228	LTCH/NH	Rehabilitation assistants, OT, PT, patient’s own GP provides clinical care	83.1 (7.1)	68	NR	NR	BI: IG 74.6 (21.7) CG 75.1 (20.9)	67.0% alone

*NR* not reported, *LTCH/NH* Long term care home/nursing home, *CPS* Cognitive Performance Scale, *CNS* clinical nurse specialist, *NP* nurse practitioner, *OT* occupational therapist, *PT* physiotherapist, *SLP* speech language pathologist, *SW* social worker, *GP* general practitioner, *SNF* skilled nursing facility, *CCI* Charlson comorbidity index, *FIM* Functional Independence Measure, *MMSE* Mini Mental State Exam, *STM* short-term memory, *CI* cognitive impairment, *ADL* activities of daily living, *IADL* instrumental activities of daily living, *BI* Barthel Index, *IG* intervention group, *CG* control group

We used TIDier checklist to abstract data on elements of the TCPs reported in the selected articles [17]. Generally, TCPs were established to reduce the demand of hospital beds and provide post-acute care including rehabilitation to older adults who were unable to be discharged home safely. Four of 37 studies specifically identified that the goal of the TCP was to offload patients from the hospital beds to wait in the TCP until admission to a LTCH. Most of TCPs were staffed by teams of health professionals, mainly physicians, nurses and physiotherapists or occupational therapists, whereas two TCPs involved only rehabilitation therapists and two were run by only nurses and general physicians. Eleven TCPs were led by nurses, out of which three were nurse practitioner (NP) led. Only eight of 37 studies reported involvement of geriatricians as part of the team. The dose was represented in the TCP length of stay, which ranged from 1 to 151 days; in all articles, the dose of specific services or treatments provided to patients was not mentioned. The components and services provided by the TCPs varied widely, details of which are provided in the processes section below (See Table 2 for the list of components). Finally, data on intervention fidelity was not presented in all articles.

**Table 2:** Transitional Care Program (TCP) Components

General category of Components	Services	Reference
1. **Assessment**	Comprehensive assessment (may include CGA)	[18, 19, 21–23, 28, 30, 33, 43, 44, 50, 52]
	Current/primary Clinical diagnosis/status	[20, 35, 38, 39, 45, 53, 55, 56]
	Mental health	[39–41, 57]
	Cognition and delirium	[37, 39–41, 57]
	Medication review	[48]
	Pain	[40, 41]
	Nutrition	[30, 37, 40, 41]
	Dental health	[57]
	Elimination	[57]
	Hearing and vision	[57]
	Functional assessment	[37, 40, 41, 48]
	Ambulation, mobility and transfer	[24, 32, 40, 41, 57]
	Falls history	[37, 40, 41]
	Confidence in coping and motivation	[48, 57]
	Assessment of social factors and supports	[48, 57]
	Review of home environment	[48, 56]
	Patients’ needs and goals assessment	[28, 33, 43, 48, 50]
2. **Care Planning and Monitoring**	Initial care plan discussed by interdisciplinary team	[19, 28, 46, 48, 50, 52]
	Weekly discussions – healthcare team members involving patients and/or family - on issues affecting participation in rehabilitation / functioning	[21, 28, 33, 37]
	Weekly discussions and updates	[23, 30, 33, 38, 42, 46, 48, 50, 57]
	Weekly revision of care plans	[28, 30, 33, 38, 42, 46, 48, 50, 57]
3. 1. **Treatment**	Continued medical care as initiated in acute care in addition to nursing, PT, SW and nutritional interventions	[18, 45]
	Nursing case management	[28, 33, 36, 37, 40, 42, 46, 49, 51, 53, 56]
	Acute, episodic medical care	[21, 23, 29, 33, 38, 44–46, 52, 53]
	Specialized interventions such as respiratory therapy, enteral nutrition, IV therapy, wound care, dialysis, pain control, terminal care	[20, 21, 24, 30, 36, 38, 40, 42, 48]
	Geriatric consultation	[37, 42, 46, 53, 55]
	Medication reconciliation	[21, 22, 38, 55]
	Mobility and rehabilitation training including transfers, stairs, strength and balance exercises and provision of mobility aids	[24, 26–30, 32, 33, 38, 40, 42, 45, 46, 48, 49, 52, 57]
	Functional training including IADL and ADL training	[24, 29, 32, 33, 40, 46, 48, 54, 57]
	Specialized rehab including SLP, hearing and dental care practitioners	[53, 54]
	Psychosocial care measures such as central dining, recreational activities, group exercises, spiritual care	[23, 24, 40, 45, 46]
4. **Discharge planning**	Multidisciplinary discharge planning	[21–24, 28, 33, 36, 38, 39, 42, 43, 52–54, 56]
	Collaboration with community partners	[43]
	Referrals/connection with exercise and social clubs	[48]
	Referrals to homecare for nursing and PT	[19]
	Referrals to homecare for nursing and personal care	[29, 49, 56]
	Discharge letter to FD	[22, 33]
	Post discharge follow up call by a nurse/PT	[19, 56]
	Post discharge follow up home visit by nurse to reinforce recommendations	[27]
5. **Patient/family & staff education**	Determine education needs of care partners; Coaching, health promotion/ safety involving caregivers before discharge	[24, 44, 46, 54, 56]
	Staff and physician education related to care of older adults and successful delivery of the TCP	[21–23, 46, 54]
	Tailored education to patient/family, related to specific medical conditions; surgical procedures; drug regimens; nutrition and food preparation; and physical activity	[30, 48]

### Structures

#### Characteristics of participants

The mean age of participants ranged from 72 [55] to 86 years [46]. The percentage of females ranged from 40% [42] to 76% [44], and of participants living alone from 26% [20] to 74% [18]. The majority of participants were functionally dependent. Most participants had mild levels of cognitive impairment; and had an average of 1.92 [56] to 8 [30] chronic illnesses.

#### Characteristics of staff

Staff working in TCPs included physiotherapists (across-studies average *n* = 26), nurses (*n* = 25), physicians (*n* = 22), occupational therapists (*n* = 20), and social workers (*n* = 17). Additional staff included dieticians (*n* = 10), geriatricians (*n* = 9), speech language pathologists (*n* = 9), pharmacists (*n* = 4); nurse practitioners (*n* = 4); clinical nurse specialists (*n* = 3); hearing services (*n* = 2); therapeutic recreation (*n* = 3); patient/health care assistants (*n* = 2); respiratory therapist (*n* = 1); and chiropodist (*n* = 1).

#### Characteristics of settings

TCPs were housed in long-term care homes (LTCH) in 12 studies, acute care hospitals in 10 studies, SNFs in five studies, community and rural hospitals in five studies, purpose-built intermediate and transitional care facilities in four studies, and multiple sites in two studies (Table 1). The TCPs were mentioned under a variety of different terms, with the most common being “Skilled Nursing Home Network Program” or “SNHN”; “University interprofessional care team” or “U-Team”; “Subacute demonstration unit”; “Transition care programs”; and “Post-acute care (PAC) facilities”, to name a few.

### Processes

#### Core components and services provided by TCPs

While the TCPs’ five core components were similar, the specific services comprising the components varied based on the main goals of the programs and the population they served. The five core components included in most TCPs were: assessment; care planning and monitoring; treatment; discharge planning; and patient, family and staff education. The specific services within each component are listed in Table 2.

##### Assessment

A variety of assessments have been identified as a core care component of TCPs in 29 of 37 studies. Fourteen studies described completing a comprehensive assessment, of which only five specifically mentioned comprehensive geriatric assessment (CGA) [18, 19, 21, 44, 50]. The majority of programs focused on more specific assessments including: function; cognition and delirium [37, 40, 41, 48, 57]; nutrition [37, 40, 41]; pain [40, 41]; confidence in coping and motivation; social supports; home environment [48, 57]; medication review [48]; dental health, elimination, hearing and vision [57].

##### Care planning and monitoring

Fourteen studies incorporated care planning and monitoring as one of the main components of TCPs. In 11 studies, the investigators reported having weekly discussions with multidisciplinary team members that in some cases included patients and/or families [21, 28, 30, 33, 37, 38, 42, 46, 48, 50, 57]. These discussions served as a means to provide patient updates and accordingly revise care plans and prepare patients and families for discharge.

##### Treatment

One of the core components offered in TCPs included treatment delivery. However, there was a wide variety of treatments offered to patients as reported in 31studies. The most frequently reported treatments were mobility and rehabilitation training including transfers, stair climbing, strength and balance exercises, and provision of mobility aids [24, 29, 30, 32, 33, 38, 40, 42, 45, 48, 49, 52, 57]. Whereas functional training involving both activities of daily living (ADL) and instrumental activities of daily living (IADL), was identified as a main treatment in seven studies [33, 41, 46, 48, 53, 57]. The next most common treatment involved acute episodic and general medical care [21, 23, 29, 33, 38, 44, 45, 52–54], followed by specialized interventions such as pain management and terminal care [20, 21, 24, 30, 36, 38, 40, 42, 48].

To support the delivery of these treatments, nursing case management [32, 36, 37, 40, 42, 46, 49, 51, 56], geriatric consultation [37, 42, 46, 53, 55], and medication reconciliation [21, 22, 38, 55] were offered. Additional psychosocial services were provided to patients such as recreational activities [23, 24, 40, 45, 46]. Interestingly, only two studies reported having specialized rehabilitation professionals deliver interventions, including speech language pathologists, as well as hearing and dental care practitioners [53, 54].

##### Discharge planning

Twenty-two of 37 studies incorporated some form of discharge planning as a core component of TCPs. Discharge planning most commonly involved multidisciplinary team members planning the individualized discharge, which was responsive to patients’ needs [21–24, 28, 36, 38, 39, 42, 52, 54, 56]. Only three of 37 studies identified completing a follow up by phone call or home visit by a physiotherapist (PT) or a nurse to reinforce treatment recommendations; as well as provide new recommendations; problem solving, counselling, support, follow up on previously initiated referrals and address issues identified by caregivers [19, 27, 56].

##### Patient, family and staff education

Of 37 studies, only 10 reported on interventions to address educational and training needs of patients, family and/or clinical staff. Authors in three studies identified that care partners’ education and training needs were assessed and addressed by various health team members, most frequently nurses and PTs [56].

In terms of supporting staff to work in TCPs, education was identified as an important element for the effective implementation and delivery of the TCP [21–23, 46, 54]. The education was provided by NPs  [21, 22], family physicians [46], and other clinicians with expertise in specialized geriatric care [23, 54]. Education included specific topics on the care of older adults, such as dementia management, medication deprescribing, as well as increasing staff’s awareness of the TCP, its goals and processes for its successful implementation [23, 54].

### Outcomes

There was a wide variety of outcomes investigated in the studies. The outcomes are meaningfully classified as patient-related outcomes and health services related outcomes. The specific categories within each of the two domains are listed in Table 3.

**Table 3:** Transitional Care Program (TCP) Outcomes

General Category of Outcomes	Components	References
**Patient Outcomes**		
**Functional status**	Functional status	[26, 29, 44, 56]
	ADL	[24, 26, 28, 30–32, 35, 36, 40, 45, 46, 48, 50, 53, 55, 56]
	IADL	[26, 30, 40, 45, 56]
**Psychological status**	Cognitive status	[30, 40]
	Psychological wellbeing	[30, 40, 45, 48, 53]
**Health Status**	Mortality	[18, 28, 29, 33, 35, 52]
	Change in disease severity	[30]
	Care needs	[28]
	Nutrition Status	[40]
	Medication Prescriptions	[21, 46]
**Quality of Life**	Quality of Life	[28, 31]
	Health-Related QoL	[45]
	Pain	[21, 40]
	Fear of falling	[50]
	Confidence to return home	[48]
**Health Services Outcomes**		
**Discharge Destination**		[18, 19, 23, 24, 27, 32, 35, 39, 40, 44, 46, 49–51, 54, 55]
**Cost Analysis**	Cost	[22, 23, 31, 35–37, 46, 51, 53]
	Healthcare Use	[33, 37, 50, 51]
	Nursing workload	[51]
	Quality of Care	[56]
	Alternative level of care	[50]
**Hospital Stay**	Length of stay	[20–23, 25, 28, 31, 33, 35, 46, 48, 50, 51, 54–56]
	Hospital readmission	[20, 21, 28, 29, 33, 50–52]
	Time to admission to long-term care	[28]
	Changes to rate of delayed discharge	[43]
	Complications during hospital stay	[46]

*ADL* Activities of Daily Living, *IADL* Instrumental Activities of Daily Living, *QoL* Quality of Life

#### Patient-related outcomes

Patient-related outcomes were classified into four categories: functional status, psychological status, health status and quality of life. These reflected patient-centered or patient-oriented outcomes measured using different instruments that were completed by patients or observers. The specific outcomes and respective measures are presented next.

##### Functional status

The functional status category is composed of functional status measures, ADL measures, and IADL measures. Functional status was measured 18 times [24, 26, 28–32, 35, 36, 40, 44–46, 48, 50, 53, 55, 56]. The most common evaluation tool was Barthel Index (BI) (*n* = 9). The majority of the studies reported improvements in functional status of patient admitted to TCPs [26, 29–32, 35, 36, 41, 44–46, 48, 54–56]. However, four studies reported no significant difference in functional status improvements between the intervention and the control groups [28, 29, 35, 56].

##### Psychological status

The psychological status category includes cognitive status and psychological wellbeing, evaluated as outcomes in 5 studies using a variety of instruments [30, 40, 45, 48, 53]. One study reported significant improvements in cognitive status of patients in transitional care units [40], and two studies reported significant improvements in psychological wellbeing [40, 45]. However, two studies reported no significant difference in improvements made in psychological wellbeing between the intervention and the control groups [30, 53], and one reported no significant change in cognitive status [30].

##### Health status

The health status category consisted of outcomes such as mortality, change in severity of disease, post discharge care needs, change in nutritional status and medication prescriptions, as listed in Table in 3. Seven studies examined mortality as a main outcome [18, 28, 29, 33, 35]. The mortality rates reported ranged from 10 to 28%. One study reported significantly lower mortality for transitional care unit patients 12 months following discharge [33]. Only one study reported significant change in disease severity from 31% of participants with low level of severity at baseline to 97% of participants at the time of discharge from the TCP [30]. One study looked at patient care needs, assessed using the Residential Care Scale, however, no significant difference in care needs was seen between the intervention and the control groups [28]. Significant positive changes in nutrition, evaluated using the Mini Nutritional Assessment (MNA), were reported in one study [40].

##### Quality of life

Quality of life measures were evaluated in three studies [28, 31, 45], whereas, two studies evaluated pain [21, 40], one examined fear of falling [50] and one explored the confidence to return home [48]. Two studies reported significant differences in quality of life of transitional care patients: one study reported that patients who were transferred to TCP had significantly lower scores on all domains of the SF-36 tool [25], and another study reported significantly greater improvements in EQ-5D scores in patients serviced by TCPs with an interdisciplinary team [49] [31, 45]. One study reported no significant changes in quality of life [28]. One of two studies that examined pain reported a significant decrease in pain [40].

#### Health services related outcomes

Health services related outcomes were grouped into three categories: 1) discharge destination; 2) health services use and cost analysis; 3) hospital stay.

##### Discharge destination

Discharge destination was measured as a main outcome in 15 studies [18, 19, 23, 24, 27, 32, 35, 39, 44, 46, 49–51, 54, 55]. More than two thirds of studies (80%) reported that the majority of patients in TCPs were discharged to community [18, 19, 23, 27, 32, 35, 39, 41, 44, 50, 51, 54]. One study reported significantly more discharges home and to assisted living facilities than to residential care facilities for patients in the intervention (i.e., exposed to TCP) group (30%), when compared to control group (12%) [46]. Only two studies reported lower chances of patients being discharged home from TCPs: patients with dementia as primary disease (24.4% discharged home) [49]; patients who had high level residential care assessments and were in a program with a lower physiotherapy staffing [24].

##### Health services use and cost

Four studies evaluated health services use for patients discharged from the TCPs [33, 37, 50, 51]; whereas 9 studies analysed costs associated with TCPs [22, 23, 31, 35–37, 46, 51, 53]. Only one of these studies examined the cost of care in relation to different types of staff mix, demonstrating that having an extra category of staff was significantly associated with lower cost [31]. Six of 9 studies found TCPs to be cost effective [22, 23, 46, 51, 53]. Studies that found TCPs to be more expensive primarily attributed the differences in cost to longer hospital stay [35, 36, 53].

##### Hospital stay

Hospital stay was examined in 20 studies [20–23, 25, 28, 29, 31, 33, 35, 40, 43, 46, 48, 50–52, 54–56]. Hospital stay was frequently assessed as length of stay prior to TCP transfer (*n* = 15 studies) and yielded mixed results. Seven studies reported TCP patients to have a significantly shorter length of hospital stay [22, 25, 28, 29, 31, 43, 54]: 6.6 days less for intervention group in one study [22], and 10.9 days less in another study [28]. Four studies showed TCP patients to have a significantly longer stay [23, 33, 35, 36]: 3.26 days more for intervention group in one study [23]; and 10.9 days more in another study [35]; and 4 studies reported no significant differences between intervention and control groups [46, 48, 50, 55]. Hospital readmission was investigated in eight studies [20, 21, 28, 29, 33, 50–52], three of which reported significantly lower rates of readmissions for patients in the intervention groups [21, 33, 52].

### Stakeholder consultation

After reviewing these findings, stakeholders identified that the TCP services incorporating mobility and strength training as well as social connection resonated with their experience. However, they also discussed the variability of TCPs based on location and resource availability. Nurses, NPs and physicians typically provided care in the TCPs, however, specialist care (e.g., geriatricians) was usually not available. In contrast to the literature, where TCPs are described as a transition point to return home, TCPs in Ontario are generally viewed as the waiting area after acute care hospitalization but before LTCH placement. In the review, TCP services were described as connecting patients to resources outside the unit; stakeholders acknowledged that, to improve patient outcomes, TCPs in Ontario should be integrated with community supports services.

When examining the TCP patient outcomes, stakeholders noted that quality of life may be more appropriate than functional outcomes for the subset of patients who are waiting for LTCH placement. However, currently, data collection is limited to mandated data such as length of stay, volume and flow of patients through the TCP. Further, as TCPs exist in a variety of settings (e.g., LTCHs, acute care hospitals, group homes, etc.) data collection is not uniform and makes comparisons across programs difficult.

Stakeholders agreed that the healthcare team staffing, resource availability, and program length, would all impact TCP patient outcomes. For example, programs staffed mainly with health care aides might focus on maintaining function whereas programs that included physiotherapists might emphasize regaining function and preventing decline. There was also discussion about refining criteria for admission to TCPs and, in doing so, distinguish suitability for TCPs from that of other programs (e.g., convalescent care, slow-stream rehabilitation, etc.) and develop processes for proactively assessing hospitalized older adults’ need for TCPs before they are designed ALC. Stakeholders identified an opportunity to establish a community of practice in this area, where approaches to defining and assessing eligibility, case management and care coordination can be shared.

## Discussion

Despite TCPs being developed globally, how TCPs are defined, funded and what services they provide vary widely [58–60]. Our review identified 37 articles describing and assessing TCPs for older adults. Within these studies, five types of programs were reported: intermediate, sub-acute and post-acute care; skilled nursing facilities and TCPs. Collectively, these programs provide short term, low intensity, restorative care to older adults who are at risk of, or designated as, delayed discharge; they are intended to promote recovery, restore independence, and provide the interface between acute care services and home [3, 8].

The papers included in this review suggest TCPs typically serve older women with multiple chronic conditions and some cognitive impairment, but who were living alone and functionally dependent before admission. This is consistent with a recent review [8] showing that these programs serve frail older adults with complex health needs, and they also found that this cohort can benefit from these services and that they can offer optimal health care services prior to transfer to the most suitable setting. Few studies reported if and how family members were involved in care and discharge planning. Given that most of these older adults were discharged home and, with limited resources in the community, family members are often involved in sustaining care once back home, this can be seen as a gap in service. Only three studies described follow-up care, either by phone call or home visit, to reinforce treatment recommendations or ongoing care (e.g., new treatments, counselling, referrals and care partner support).

Our review delineated common core components that have been integrated in TCPs internationally, including assessment, care planning, treatment, discharge planning, and education. However, the variability in the specific services offered within each component was interesting given that the programs had similar purposes. In terms of assessments, comprehensive geriatric assessments are the gold standard [61] when caring for older adults with multiple comorbid conditions yet only four TCPs were found to use them. Given the population served by TCPs, further research should focus on the benefits of integrating the comprehensive geriatric assessments and a more holistic approach to care.

The individual services offered in the treatment component of TCPs were also highly variable and were specific to the goals of and the population targeted by the program within the health care organization. The most frequently reported treatment focused on rehabilitation and included a focus on transfers, stair climbing, strength, and balance exercises. These activities are essential to regain function [9] and are foundational in TCPs. Missing from many of TCPs were services focused on patients’ social and mental health needs. Social vulnerability stemming from the accumulation of multiple and varied social problems, such as inequities , loss of social support and networks and socioeconomic status is a concern, in part because of the impact on health [62]. Future studies of TCPs should account for these social vulnerabilities, including in planning for discharge.

Staff delivering the intervention were of diverse disciplines and the staff mix or complement varied according to the specific treatments delivered. The most frequently cited staff working in the programs were physiotherapists and nurses. A review of intermediate models of care suggest that care should be delivered by an interdisciplinary team within an integrated health and social care system where a single contact point optimises services access, communication and coordination [8]. Notably absent from the teams were hearing and vision specialists; given the increasing number of older adults and age-related increased risk of dual sensory loss and its associated challenges [63], more screening and services are required.

As reported in the articles reviewed, the TCPs share goals similar to those of geriatric rehabilitation (GR) programs, including to improve functional capacity, promote activity and preserve functional reserve and societal participation [64]. We advocate the integration of the core principles of GR that also utilizes reablement approach [65] in the design of TCPs to enhance patient outcomes, in addition to refinement to the staff complement and processes currently in use in existing TCPs. Reablement approach not only focuses on improving functional abilities but also on maintaining current abilities and independence. Accordingly, TCPs should entail: 1) provision of person-centered care where the patients and care partners are actively involved in goal setting and care planning taking into account psychosocial and environmental factors. If older adults have reached a stage where they have lost their ability to participate in advance care planning (ACP) and goals of care discussions due to advanced stages of neurodegenerative diseases, their care partners should be engaged in such conversations while keeping the patients’ best interest at the core. Furthermore, goals should be operationalized with an appreciation of person’s abilities so that they are achievable, realistic and meaningful for patients and care partners [65]; 2) conduct of comprehensive geriatric assessments which incorporate the perspectives of patients as well as care partners to help tailor rehabilitation interventions to patients’ physical and social needs; 3) involvement of interdisciplinary team members (nurses, PT, OT, SLP and social workers) and clinicians who have training and experience in care of older adults with in-house ready access to providers such as NPs and/or geriatricians to assess and treat acute changes in conditions*;* 4) use of strategies utilizing restorative and compensatory methods [65]. TCPs are sometimes used to offload older adults from the hospital beds to another less expensive care setting without providing the care that is required, demanding careful attention to potential misuse of these programs [59]. Therefore, a careful review of the patients’ goals of care must be articulated in acute care prior to admission to the TCP to ensure patients are in the best location.

There was significant variability in the patient-level outcomes measured to evaluate the impact of TCPs, including functional, cognitive, quality of life and health status measures. Health system outcomes included discharge destination, costs and hospital stay (length of stay, readmission rates). These results are consistent with a recent review that found similar outcomes being used to evaluate ICMs [9]. Our review corroborates the finding that comparisons between various TCPs may be difficult given the multiple outcomes used to evaluate them [8, 9]. Moving forward, we emphasize the importance of clarifying the conceptualization of TCPs and mapping the outcomes to the TCP components; this would facilitate the identification of core outcomes that should be carefully and consistently operationalized to enable meaningful comparisons across TCPs within and across countries.

It is about time we pose the question, why do we continue to build TCPs that are often related to restoring excess disability that was caused by acute care? Is it not time to focus on improving the hospital experience for older adults, especially those with dementia? Based on a review of acute care practices [66], ageism exists and better acute care practices can occur upstream instead of focusing on repairing losses that occurred in hospitals. A focus on better home care options [67] may also reduce the need for these ever-increasing TCPs which are currently being developed worldwide. TCPs, regardless of their name or design should not serve as holding places for these complex, frail and vulnerable individuals waiting for LTC admission, but should include staff who can work with older adults to attain their unique and full restorative potential in maintaining their independence if there is potential to do so, while also meeting their holistic and social care needs which may include helping them come to terms with their lost function and end of life. The strengths of the scoping review include a comprehensive and systematic search of the literature which identified factors that may influence the implementation and outcomes of TCPs in addressing delayed hospital discharges for older adults. Still, there were limitations; in particular, some relevant articles may have been excluded by limiting the search to four languages.

## Conclusion

TCPs can influence outcomes for hospitalized older adults, including helping them go back home. This review highlights the need to design TCPs to be delivered by interdisciplinary teams, accommodating persons with cognitive impairment and actively involving care partners. Clinicians, administrators and policymakers should collaborate to establish TCPs that proactively admit older adults at risk for delayed discharge, focus on optimizing functional ability, and incorporate individualized care planning and care coordination. Additional studies are required to investigate the contributions of TCPs within integrated health care systems.

## Supplementary Information

**Additional file 1.** Medline search strategy.

## Data Availability

All data analysed during this study are included in this published article.

## Abbreviations

TCP: Transitional care program
QoL: Quality of life
ALC: Alternate level of care
ICMs: Intermediate care models
SNFs: Skilled nursing facilities
ScR: Scoping review
MeSH: Medical subject headings
SNHN: Skilled nursing home network
PAC: Post-acute care
CGA: Comprehensive geriatric assessment
ADL: Activities of daily living
IADL: Instrumental activities of daily living
PT: Physiotherapist
BI: Barthel Index
MNA: Mini nutritional assessment
LTCH: Long term care home
MCC: Multiple comorbid conditions
