# Supplementary material for: Understanding transitional care programs for older adults who experience delayed discharge: a scoping review
Source: BMC Geriatr. 2021 Mar 29;21:210. doi: 10.1186/s12877-021-02099-9 (PMC8008524; doi:10.1186/s12877-021-02099-9)
Supplement: Supplementary file 1 — Additional file 1. Medline search strategy. [file 12877_2021_2099_MOESM1_ESM.docx]

Supplement A. Medline (OVID) Search Strategy

1. alternat* level? of car*.tw,kf.

2. (delayed adj2 discharge?).tw,kf.

3. (bed adj (block? or blocking or blocker?)).tw,kf.

4. ((convalescen* or restorative) adj (unit? or bed? or care or program* or ward? or facility or facilities or setting?)).tw,kf.

5. ((subacute or sub-acute) adj (unit? or bed? or care or program* or ward? or facility or facilities or setting?)).tw,kf.

6. Subacute Care/ [Mesh definition: Medical and skilled nursing services provided to patients who are not in an acute phase of an illness but who require a level of care higher than that provided in a long-term care setting.]

7. ((postacute or post-acute) adj (unit? or bed? or care or program* or ward? or facility or facilities or setting?)).tw,kf.

8. (transition* care adj (unit? or bed? or care or program* or ward? or facility or facilities or setting?)).tw,kf.

9. ((stepdown or step-down) adj (unit? or bed? or care or program* or ward? or facility or facilities or setting?)).tw,kf.

10. (intermediate adj (unit? or bed? or care or program* or ward? or facility or facilities or setting?)).tw,kf.

11. Intermediate Care Facilities/ [Mesh definition: Institutions which provide health-related care and services to individuals who do not require the degree of care which hospitals or skilled nursing facilities provide, but because of their physical or mental condition require care and services above the level of room and board.]

12. "complex continuing care".tw,kf.

13. or/1-12

14. exp aged/

15. Geriatrics/ or Geriatric Assessment/

16. (geriatri* or gerontol* or elder? or elderly or elderlies or senior* or senescen* or septuagenarian* or octogenarian* or nonagenarian* or pensioner*).tw,kf.

17. ((old* or aged*) adj5 (adult? or people or patient? or person? or wom#n or m#n or individual? or client?)).tw,kf.

18. "oldest old".tw,kf.

19. "old-old".tw,kf.

20. or/14-19

21. Health Services, Indigenous/

22. exp Ethnopharmacology/

23. exp american native continental ancestry group/

24. indians, north american/

25. inuits/

26. Oceanic Ancestry Group/

27. (Athapaskan or Saulteaux or Wakashan or Cree or Dene or Inuit or Inuk or Inuvialuit* or Haida or Ktunaxa or Tsimshian or Gitsxan or Nisga'a or Haisla or Heiltsuk or Oweenkeno or Kwakwaka'wakw or Nuu chah nulth or Tsilhqot'in or Dakelh or Wet'suwet'en or Sekani or Dunne-za or Dene or Tahltan or Kaska or Tagish or Tutchone or Nuxalk or Salish or Stl'atlimc or Nlaka'pamux or Okanagan or Sec wepmc or Tlingit or Anishinaabe or Blackfoot or Nakoda or Tasttine or Tsuu T'inia or Gwich'in or Han or Tagish or Tutchone or Algonquin or Nipissing or Ojibwa or Potawatomi or Innu or Maliseet or Mi'kmaq or Micmac or Passamaquoddy or Haudenosaunee or Cayuga or Mohawk or Oneida or Onodaga or Seneca or Tuscarora or Wyandot).tw,kf.

28. aborigin*.tw,kf.

29. Indigenous*.tw,kf.

30. Metis.tw,kf.

31. red road.tw,kf.

32. "on reserve".tw,kf.

33. off-reserve.tw,kf.

34. First Nation?.tw,kf.

35. Amerindian.tw,kf.

36. maori*.tw,kf.

37. eskimo?.tw,kf.

38. (native* adj (alask* or american* or canadian* or hawaii)).tw,kf.

39. (pacific adj islander*).tw,kf.

40. (american adj (indian* or samoan*)).tw,kf.

41. (torres adj strait adj islander*).tw,kf.

42. tribe?.tw,kf.

43. (urban adj3 (Indian* or Native* or Aboriginal*)).tw,kf.

44. ethnomedicine.tw,kf.

45. or/21-44 [cite: Campbell, Sandy, Marlene Dorgan and Lisa Tjosvold. Filter to Retrieve Studies Related to Indigenous People of Canada the OVID Medline Database. John W. Scott Health Sciences Library, University of Alberta. Rev. March 8, 2016. http://guides.library.ualberta.ca/ld.php?content_id=14026803 ]

46. 13 and 20

47. 13 and 45

48. 46 or 47

49. 48 not ((exp infant/ or exp child/) not exp adult/)

50. 49 not (exp animals/ not exp humans/)

51. (dutch or english or french or german).lg.

52. 50 and 51
